# Microwave-Assisted Brine Extraction for Enhancement of the Quantity and Quality of Lipid Production from Microalgae *Nannochloropsis sp*.

**DOI:** 10.3390/molecules24193581

**Published:** 2019-10-04

**Authors:** Nour Zghaibi, Rozita Omar, Siti Mazlina Mustapa Kamal, Dayang Radiah Awang Biak, Razif Harun

**Affiliations:** 1 Department of Chemical and Environmental Engineering, Faculty of Engineering, University Putra Malaysia, 43400 Serdang, Selangor, Malaysia; dradiah@upm.edu.my (D.R.A.B.); mh_razif@upm.edu.my (R.H.); 2 Department of Process and Food Engineering, Faculty of Engineering, University Putra Malaysia, 43400 Serdang, Selangor, Malaysia; smazlina@upm.edu.my

**Keywords:** Microwave-assisted Extraction, brine, lipid, microalgae, polyunsaturated fatty acids

## Abstract

Toward attaining a sustainability and eco-friendly process, a green and low-cost solvent—brine (NaCl solution) is proposed, as microwave-assisted extraction (MAE) technique solvent to extract lipids from microalgae *Nannochloropsis sp*. The effect of NaCl concentration on the quantity and quality of the extracted lipid was assessed, while MAE parameters were optimized using response surface methodology (RSM). The content of fatty acid methyl esters (FAMEs) in the lipid was analyzed by using a gas chromatography—flame ionization detector (GC/FID). The highest lipid yield (16.1%) was obtained using 10% (*w*/*v*) brine at optimum extraction parameters of 5% (*w*/*v*) solid loading, 100 °C, and 30 min. The lipid extraction yield via optimized MAE-brine technique was thrice better than that Soxhlet extraction did and only 2% less than Bligh and Dyer (B&D) lipid extraction, which utilized harmful solvents. The proposed MAE-brine technique offered better quality lipids containing the highest amount of polyunsaturated fatty acids (PUFA) (44.5%) and omega-3 fatty acids (FAs) (43%). Hence, the MAE-brine solvent technique appears to be a promising extraction method for cheaper, greener, and faster extraction of a high-quality lipid for specialty food applications.

## 1. Introduction

Microalgae compositions have been a discriminatory food supplement source, as it meets public health and dietary intake requirements complete with protein, carbohydrate, and lipid [1]. *Nannochloropsis sp.* is a genus microalga, in which the taxonomic classification was first termed by Hibberd (1981) [2]. It has been considered as a good candidate for fatty acid (FA) natural source, as it contains high lipids, when compared to other types of microalgae. Table 1 presents the composition of a range of microalgae.

Fatty acids (FAs) are the main composition of fats or oils, which are formed by hydrophobic aliphatic chain and hydrophilic carboxylic group. Depending on their aliphatic chain nature, FAs are categorized into saturated FA (SFA), monounsaturated FA (MUFA) and polyunsaturated FA (PUFA). PUFAs are long chain FAs that contain more than two double bonds on their aliphatic chain [5]. Its derivatives, such as omega-3 and omega-6, are essential FAs that play a vital rule in terms of growth and repair in human body. Nevertheless, humans are unable to synthesize these FAs in their bodies, hence the consumption through diet or supplements. Omega-3 includes three main FAs: α-linolenic acid C18:3 (ALA), eicosapentaenoic acid C20:5 (EPA) and docosahexaenoic acid C22:6 (DHA) [1]. A number of health organizations have recommended that the daily dose for adults must be between 250 and 500 mg of EPA and DHA for their positive effect on brain development, heart disease prevention, and vision health protection [6].

Fish and seafood products are the main commercial source for EPA and DHA production [7]. Nonetheless, some adversities of consuming fish as a source of FAs are due to open to risk of chemicals contamination, unpleasant taste and odor, and unacceptable amongst vegetarians [8]. Such shortcomings have led to the discovery of an alternative source. Microalgae has been considered as the main source of omega-3 PUFAs, wherein other marine organisms rely on it to obtain EPA and DHA through bioaccumulation [9].

Although microalgae are rich in essential FAs such as omega-3, several main steps are involved in its lipid production namely cultivation, harvesting, and extraction of the lipid, and later, purification of FAs. Among these steps, the lipid extraction stage is a crucial step to enhance the quality and quantity of microalgal lipid production [10]. Various extraction methods have been proposed for this purpose, both conventional and non-conventional extraction techniques. The conventional techniques commonly involve chemical extraction methods that depend on diffusion of organic solvents through microalgae cells, such as hexane or hexane/isopropanol mixture via Soxhlet extraction [11], chloroform/methanol in Folch [12] and Bligh and Dyer methods (B&D) [13]. On the other hand, the non-conventional techniques depend on disruption of microalgae cell wall using mechanical equipment such as bead mills [14] and expeller press [15]. Other non-conventional methods are usually coupled with solvent extraction, for instance, microwave-assisted extraction (MAE) with chloroform/methanol [16] or with a mixture of isopropanol/hexane [17] and ultrasound-assisted extraction with hexane or methanol [18]. This method also embeds enzymes prior to hydrolysis of microalgae cell wall [19].

Although some of the abovementioned methods have many advantages, such as high extraction efficiency and energy-effective [15], most of the methods are not environmentally friendly due to use of toxic solvents. Others such as supercritical fluid extraction are more environmentally friendly, but dealing with a high extraction pressure resulted in higher energy consumption [20]. In addition, some methods display low yield due to extended extraction time, as well as cost ineffective due to high solvent and expensive enzyme usages.

Many studies have reported that the MAE method offers vast advantages over conventional techniques, as the process is non-intricate and environmentally friendly [21,22], minimal use of solvent [23], lower operating cost and extraction time [24]. Moreover, the MAE technique showed a higher lipid extraction efficiency in comparable with non-conventional methods, see Table 2.

Some factors that affect MAE efficiency include temperature, time, solid loading, solvent type, and its dielectric properties [31,32]. The microwave heating principles have been reported previously by many studies [33,34,35]. These studies reported that the microwave heating method heavily depends on the effect of electromagnetic field on the molecules by dipole rotation and ionic conduction. The dipole rotation heating principle refers to the rearrangement of dipole molecules in electromagnetic field, while the ionic conduction works with migration of ions when the field is applied. 

In order to generate a rapid heat-up through microwave radiation, the solvent should interact with electromagnetic radiation and possess high efficiency to absorb microwave energy to be converted into heat [36]. The success of these phenomena can be increased with dielectric properties (ε*) of the solvent, which is defined by two parameters expressed in Equation (1) [37].

**Figure 1:**
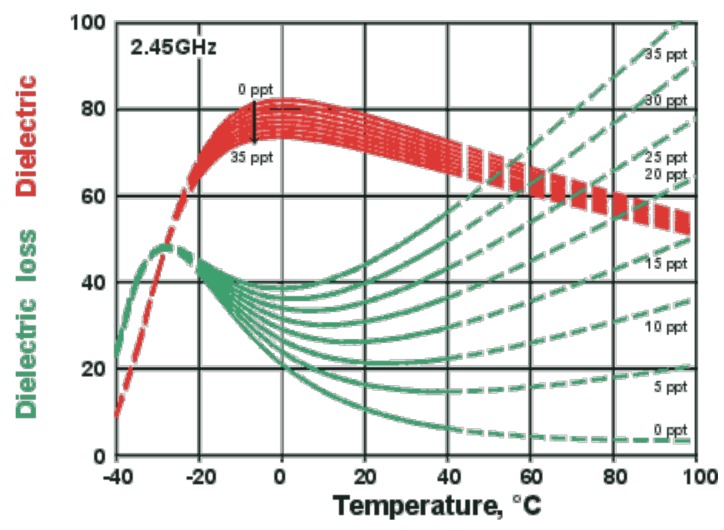
The effect of salt concentration on dielectric constant and dielectric loss under microwave irradiation [41].

ε* = *ε*′ − *jε*″ (1)

where, *j* = $\sqrt{-1}$, *ε*′ is solvent dielectric constant that measures the ability of a material (solvent in this case) to absorb microwave radiation. *ε*″ denotes the imaginary factor or the material dielectric loss, which is defined as the efficiency of a material in converting microwave energy into heat. The ratio of dielectric loss to constant called loss tangent (tan δ) describes the energy dissipation factor. Both *ε*′ and *ε*″ are 70 and 13 for water, respectively, and tan δ of 0.1 at 25 °C makes water a good candidate as microwave solvent. [37,38] studied the presence of inorganic salt in a polar solvent and its effect on dielectric loss, which enhanced under microwave irradiation (see Figure 1). This phenomenon is due to the fact that the presence of ions may increase solvent conductivity and affect heating rate [39], attributable to the dual effect of Joule heating and dipolar relaxation [40]. Joule heating refers to the passage of an electric current through a conductor that produces heat.

Conductivity affects loss of solution, which can be represented by σ_i_ and σ_d_ for Joule heating and dipolar relaxation, respectively, while the corresponding loss factors are denoted by *ε_i_*″ and *ε_d_*″. The correlation between dielectric loss and conductivity is portrayed in Equation (2).

*ε*″ = (*σ_d_* + *σ_i_*)/*ωε_o_* (2)

where *ω* stands for angular frequency of electromagnetic wave. Upon combining Equations (1) and (2), as well as substituting *σ_i_* and *σ_d_* with *ε_i_*″ and *ε_d_*″, respectively, the yield is as given in Equation (3).

ε* = *ε*′ − *j* (*ε_d_*″ + *ε_i_*″)/*ωε_o_* (3)

when salt concentration increases, *ε_i_*″ contribution predominates over *ε_d_*″, thus increasing the value of *ε*″. This means; higher value of dielectric loss increases the dielectric heating rate.

The addition of salt changes the natural structure of water, as it reduces the dielectric constant value. The effects of salt concentrations under microwave irradiation were tested by Meissner and Wentz [41]. As illustrated in Figure 1, the temperature has a negative impact on the dielectric constant value. This is because; as temperature increases, the strength and extent of hydrogen bonding decrease. The effect of salt concentration on dielectric loss is more significant at higher microwave temperature. More ions are bound to enhance the Joule heating process, thus resulting in higher loss value.

This paper demonstrates the feasibility of MAE as an ecofriendly method for lipid extraction from *Nannochloropsis sp.* microalgae by using a green inorganic salt (NaCl) solution as the extraction solvent. The effect of salt concentration on the extraction yield was investigated, along with several extraction parameters; solid loading, temperature, and time, which were optimized using Response Surface Methodology (RSM). The fatty acid methyl esters (FAMEs) resulted from lipid transesterification were analyzed using gas chromatography equipped with a flame ionization detector (GC/FID) for lipid qualification. *Nannochloropsis sp.* microalgae was the strain selected for this work as it exerts exceptional potential PUFAs source, especially EPA [42].

## 2. Results and Discussion 

### 2.1. Conventional Extraction 

The B&D method is an established and rapid method for lipid extraction, while Soxhlet is a commonly used method for most extraction due to its high solvents′ acceptability depending on the product of interest. In this study, B&D and Soxhlet methods were employed as the comparative analyses to MAE method. The outcomes of lipid extraction for both conventional methods indicated that the B&D method extracted four times more lipids at 18%, in comparison to only 4.5% extracted via Soxhlet method. Despite the lengthy extraction process, the Soxhlet method failed to give better extraction yield.

The B&D method resulted in higher FAMEs (38.65 mg/g microalgae) than did the Soxhlet method (11.48 mg/g microalgae). Additionally, the B&D method appeared to be superior to the Soxhlet method when extracting PUFAs and omega-3 FAs at 10.4 mg/g (26.9%) and 8.25 mg/g (21.3%), respectively. The PUFAs and omega-3 FAs obtained from the Soxhlet method were only 0.8 mg/g (7.07%) and 0.05 mg/g (0.46%), respectively. 

The high extraction yield generated by the B&D method was due to the ability of the solvent mixture (chloroform/methanol) to extract all types of microalgae lipids (polar and neutral lipids). The non-polar hexane solvent has been reported to display selectivity toward lipids with low polarity as it is used mainly to extract neutral lipids [43]. This also explains the ability of the B&D method on extracting higher number of omega-3 FAs and PUFAs, which is usually noted in polar microalgal membrane lipids that is difficult to extract using non-polar solvents, such as hexane [44]. Other solvents were not attempted as the focus of this study is narrowed toward seeking a greener solvent for lipid extraction from *Nannochloropsis* microalgae.

### 2.2. Effect of Inorganic NaCl Salt Concentration on Quantity and Quality of Lipids Using MAE

#### 2.2.1. Lipid Extraction 

**Figure 2:**
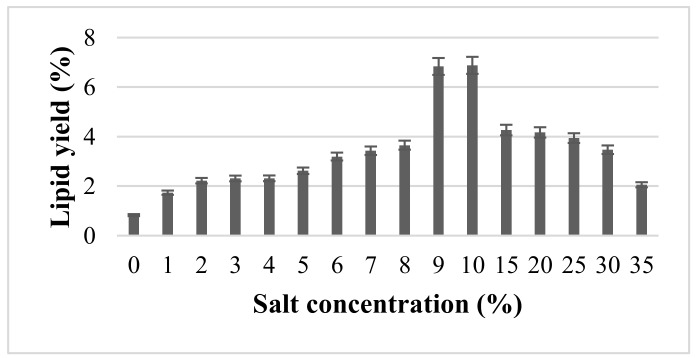
The effect of different NaCl salt concentrations on the extracted lipid using microwave-assisted extraction (MAE) at 10% (*w*/*v*) solid loading, 100 °C and 5 min.

MAE utilizing salt solution as the solvent was employed to assess the yield of lipid. Salt solution at 1–35% (*w*/*v*) concentrations had been applied and the outputs are shown in Figure 2. The MAE was run at 10% (*w*/*v*) solid loading, 100 °C and 5 min. A control study using distilled water was performed, denoting 0% salt concentration. The figure shows that the presence of salt had a positive impact on lipid yield, while the absence of salt generated the lowest yield (<1%). The yield of lipid increased with increment in NaCl concentration up to 10%, with 6.88% exerting the highest yield, but reduction in yield with increasing salt concentration.

The effect of inorganic salt on enhancing microwave absorption and reaction rate was investigated by [45]. They claimed that the existence of ions (from the salt) in polar solvent, such as water, exemplified a great effect on dielectric loss, which is responsible for dissipating heat and raising temperature at a rapid rate. As portrayed in Figure 1, higher salt concentration resulted in higher dielectric loss due to higher conductivity from the ions present in the solution, which dramatically influenced the heating rate [39]. At certain salt concentration, the cell wall may have cracked, thus resulting in abrupt high increment of lipid yield. This is attributable to the superheating effect that led to 10–30 °C in excess of the normal boiling point of water [46].

Additionally, the demulsification effect during recovery of lipid could have enhanced the yield of lipid upon increment in salt concentration. Prior studies confirmed that extraction of microalgal lipids in the presence of high amount of water or other contacts, such as protein, hindered the extraction and decreased the FAs recovery, as a result of formation of oil/water emulsion [4]. Therefore, many studies have applied the combination of microwave and salt solution to demulsify oil/water emulsion [47,48,49]. Increase in salt concentration enhanced the demulsification process, primarily because increased ions lead to better superheating characteristic and better electric effect [39].

Further increment of salt concentration before 10%, nonetheless, did not result in higher lipid yield. Thus, it has been hypothesized that there is a limit at which only certain salt concentrations can be beneficial for demulsification of oil/water emulsion. [39] assessed the impact of incorporating NaCl salt on the formation of water into crude oil during petroleum production. As a result, a significant effect of NaCl concentration as a demulsifier was noted up to 0.04 mol L^−1^, wherein increased NaCl concentration further restabilized the emulsion. It is believed that the demulsification process is an integration of various influential factors, in which salt concentration is one of them. [50] explained the effect of increasing salt concentration on emulsion stabilization, wherein higher salt concentration resulted in lower interfacial tension between oil and water, which facilitated the formation of small oil droplets. The small oil droplets stabilized the oil/water emulsion. In a study related to crude oil formation in water pipelines, [51] found similar agreements and postulated that the increase in salt concentration can stabilize oil/water emulsion due to the presence of ions that may serve as barriers between oil droplets and water. Overall, increment in salt concentration enhanced emulsion stability.

#### 2.2.2. Fatty Acid Composition 

**Figure 3:**
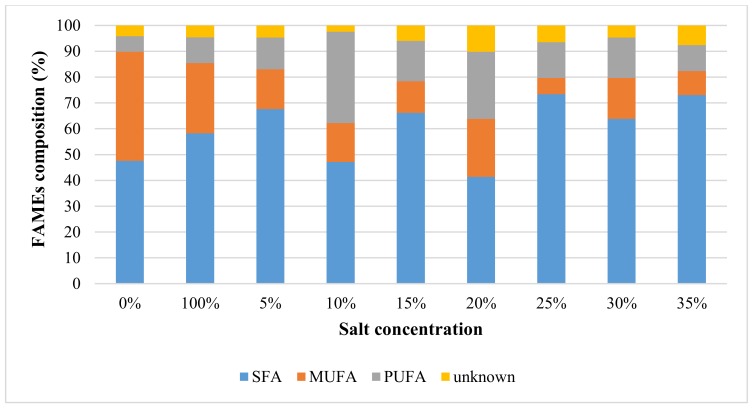
The distribution analysis of FAMEs classes for *Nannochloropsis sp*. microalgae using MAE technique at constant extraction parameters of 10% (*w*/*v*) solid loading, 100 °C and 5 min with different salt concentrations ranging between 0 and 35%.

Table 3 presents the yield (mg/g of microalgae) for 20 FAMEs of *Nannochloropsis sp.* microalgae at varied salt concentrations using MAE technique at 10% (*w*/*v*) solid loading, 100 °C and 5 min. The most abundant compound among the 20 FAs was palmitic acid (C16:0) and followed by EPA (C20:5). Salt concentration of 10% (*w*/*v*) gave the highest PUFAs value of 10.42 mg/g, which exceeded 50-fold of PUFAs yield recorded for extraction without salt (0.18 mg/g). For most of the extracts, SFAs appeared to be dominant, followed by PUFAs and MUFA (see Figure 3). The percentage of PUFAs ranged between 6 and 35%, depending on the salt concentration, in which the highest was found at 10% (*w*/*v*) NaCl concentration. Concurrently, the 10% salt concentration gave the highest omega-3 distribution, where the EPA was the most abundant omega-3 compound. 

Lipid extraction from *Nannochloropsis sp.* microalgae using MAE-brine (10% (*w*/*v*) salt concentration) at parameters of 10% (*w*/*v*) solid loading, 100 °C and 5 min, had been compared to the results retrieved from conventional methods. Despite the higher lipid recovery (6.88%) than Soxhlet (4.5%), the proposed MAE seemed to be more than twice less efficient than B&D (18%). The lower yield found for MAE-brine technique, in comparison to B&D method, calls for optimization of microwave parameters.

The high lipid extraction using B&D method signifies the ability of its solvents to extract all types of lipids via diffusion [43]. Meanwhile, the lower extraction yield obtained using the Soxhlet method reflects its limited ability to diffuse polar lipids [43], in addition to failure in disrupting the hard microalgal cell walls, such as *Nannochloropsis sp.* microalgae. King et al. [52] revealed full disruption of *N. salina* cells at 16 min of ultrasound extraction, while *Nannochloropsis* cells were barely affected. Cravotto et al. [53] reported that MAE using hexane solvent yielded more than thrice lipid from marine *Crypthecodinium cohnii* microalgae, when compared to that retrieved using Soxhlet extraction method.

Many studies [10,27,54] evaluated the effectiveness of MAE in disrupting microalgae, which can affect lipid yield recovery. For example, Lee et al. [10] found that MAE is the most efficient method for lipid extraction from *Botryococcus sp*., *Chlorella vulgaris* and *Scenedesmus sp.* microalgae, in comparison to sonication, autoclaving, and bead beating methods. 

Both total FAMEs recovery and FAMEs distribution analyses for MAE-brine (10% (*w*/*v*) salt concentration), which displayed the highest lipid and PUFAs extracted, had been compared with the outcomes obtained from the conventional methods of Soxhlet and B&D. Although the B&D extraction method yielded the highest lipid, the FAMEs compositions signified that both methods (B&D and MAE-brine) extracted similar amount of PUFAs and Omege-3 approximately 10 mg/g and 8 mg/g, respectively.

**Figure 4:**
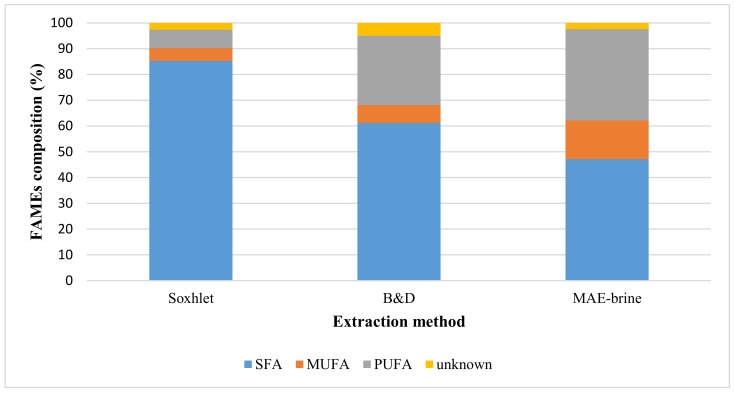
The distribution analysis of FAMEs classes for *Nannochloropsis sp*. microalgae for three extraction methods of Soxhlet, B&D and MAE-brine technique at extraction parameters of 10% (*w*/*v*) solid loading, 100 °C and 5 min.

Figure 4 illustrates the percentage distribution of FAMEs classes, where it clearly showed that MAE-brine technique (at extraction parameters of 10% (*w*/*v*) solid loading, 100 °C and 5 min) extracted the most PUFAs at 35.4%, followed by B&D at 26.9% and finally the Soxhlet method that yielded only 7.07%. As for the percentage of extracted omega-3, the highest outcome was recorded for MAE-brine, when compared to B&D and Soxhlet at 29.0%, 21.3% and 0.5%, respectively. The low PUFA extracted in Soxhlet could be due to the solvent hexane that can only extract non-polar FAs or neutral lipids from microalgae cells. The mixture of chloroform and methanol used in the B&D method had the capability to extract polar and non–polar lipids from microalgae cell [55]. On the other hand, but the studies by other researchers had confirmed that the microalgae used in this study (*Nannochloropsis* sp.) has greater amount of polar lipids than neutral lipid. For example, the study of Yao et al. [56] found a more than double amount of polar lipids than non-polar lipids.

The results from conventional extraction are in agreement with that reported previously [57]. Polar solvents (chloroform and methanol) used in B&D extraction displayed more ability in extracting polar lipids from microalgae cell membrane, such as PUFAs. The Soxhlet extraction with non-polar solvent (hexane) exhibited more tendency towards neutral lipid extraction, thus resulting in the Soxhlet method extracting more SFAs than PUFAs.

Water, as a highly polar solvent (dielectric constant = 80 at 20 °C), has more ability to absorb microwave energy, along with the presence of salt that can improve the dielectric loss responsible for converting microwave energy into heat. These two factors can improve the efficiency of microwave extraction, thus increasing microalgae cell disruption that has relative influence on PUFAs production, which is considered the main block of microalgal polar lipids (glycolipids and phospholipids) [44]. The low extraction time of MAE can preserve the thermolabile compounds, such as PUFAs, from degradation at higher temperature [23].

### 2.3. Optimization of Lipid Yield Using RSM

#### 2.3.1. Lipid Extraction Optimization 

The RSM utilizing CCD experimental design was performed to assess the effects of microwave parameters, namely solid loading (A), microwave temperature (B) and extraction time (C), on lipid extraction (Y). Coded and actual experimental runs, as well as retrieved response (Y), are listed in Table 4. The highest extraction yield was recorded at 10% (*w*/*v*) solid loading, 90 °C and 25 min of extraction time (experimental runs: 10). The obtained regression equation for lipid recovery (Y) is as follows:

Y = 34.34117 − 0.85955 × A − 0.64600 × B − 0.54280 × C − 6.16175E^−3^ × A × B + 3.81567E^−3^ × A × C + 3.38117E^−3^ × B × C + 0.032721 × A^2^ + 5.49845E^−3^ × B^2^ + 8.25710E^−3^ × C^2^ (4)

**Figure 5:**
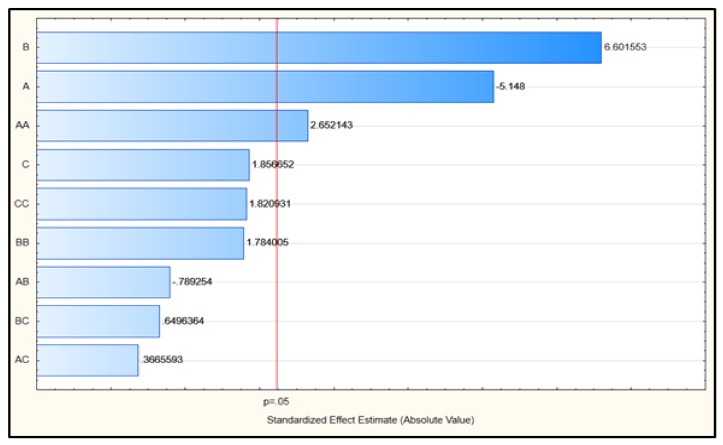
Standardized effect of Pareto chart. A = solid loading, B =extraction temperature and C = extraction time.

In explaining the extent of influence for each coefficient on the response factor, the Pareto chart (see Figure 5) had been used. The length of each bar estimates the influence of each parameter. The level of significance is defined by the vertical line with a confidence of 95%. The microwave extraction temperature (B) emerged as the most significant factor, and followed by microwave solid loading (A).

Table 5 presents the ANOVA analysis for CCD results; the statistical significance for the model is indicated by a *p*-value of 0.0002. The squared regression value (R^2^) of 0.9227 reflected that 92.3% of the CCD data could be explained by the model. This shows that the model represents a real correlation between the coefficients. The ANOVA results signified the statistical significance for each coefficient, whereby two linear coefficients (A,B) and an interaction coefficient were statistically significant (*p*-value < 0.05), whereas the other coefficients were statistically insignificant (*p*-value > 0.05). The significant coefficients displayed a noticeable impact on lipid extracted using MAE, while insignificant coefficients had less or neglected the influence, depending on their *p*-values. Accordingly, the predictive equation given in the following was acquired after omitting most of the insignificant coefficients:

Y = 36.00002 − 1.28571 × A − 0.67925 × B − 0.21507 × C+ 0.032721 × A^2^ + 5.49845E^−3^ × B^2^ + 8.25710E^−3^ × C^2^ (5)

**Figure 6:**
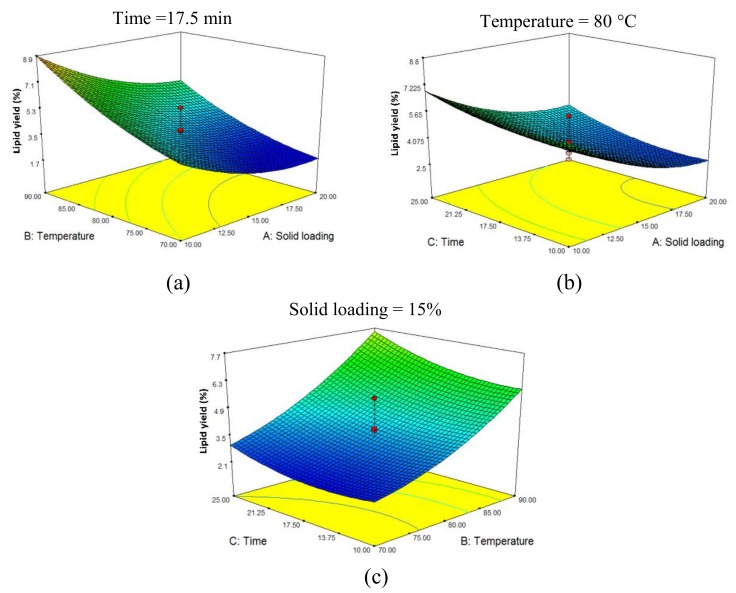
Response surface plots displaying the effect of (**a**) microwave solid loading and extraction temperature, at Time = 17 min (**b**) microwave solid loading and extraction time, at Temperature = 80 °C and (**c**) the extraction temperature and time at Solid loading = 15% on lipid extraction yield.

In comprehending the interaction between microwave extraction parameters, and in determining the optimum level for the maximum extracted lipid from *Nannochloropsis sp.* biomass, the 3-D response surface plots were developed, as portrayed in Figure 6a–c. Each plot relates two factors, while the third factor is fixed at its medium value (central point). As discussed in the above, both microwave extraction temperature and solid loading displayed the most influence on lipid yield. Lower solid loading, as well as higher extraction temperature and time, resulted in higher lipid yield extraction. The model offers maximum lipid yield for 5% (*w*/*v*) solid loading, a temperature of 100 °C and 30 min of extraction time. The predicted value of lipid yield from the model was 18.4%, while the empirical value for the said conditions was 16.1%. The MAE-brine that applied values derived from the model successfully yielded higher lipid, which is comparable to the conventional extraction methods, such as the B&D method that generated 18%. The MAE is ecofriendly as it only uses water and NaCl (table salt) to extract lipid, whereas the solvents used in Soxhlet and B&D methods are not only costly, but also toxic to humans and the environment. 

#### 2.3.2. Fatty Acid Analysis for Optimization Experiment

**Figure 7:**
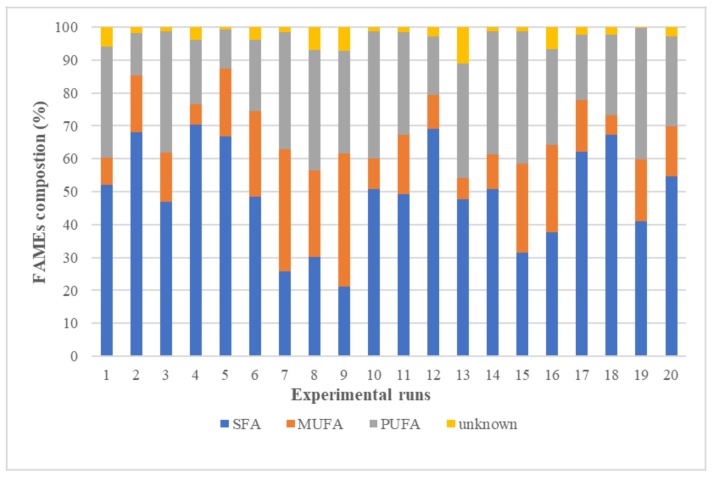
The distribution analysis for the experimental design including FAMEs classes′ percentages obtained from *Nannochloropsis sp*. microalgae using MAE technique with 10% (*w*/*v*) brine as the extraction solvent.

The compositions of lipid obtained from the 20 runs in RSM experiment were determined by using GC-FID. The results for the 20 FAs were analyzed and classified into classes (see Figure 7). It is apparent that the compositions of FAME varied according to the experimental conditions. Most of the experimental runs had a majority of either PUFAs class or SFAs class, while a minority for MUFAs class. The best PUFAs percentages of 40.2%, 39.9% and 38.5% were given by runs 15, 19 and 10, respectively, with the common denominator among these experiments was the high extraction temperature of 100 °C and 90 °C and the long extraction time of 25 min for runs 10 and 19. The results indicated that the PUFAs fraction could be affected by microwave extraction temperature and time. 

In order to confirm the effect of extraction temperature on the percentage of extracted PUFAs, the results for 5th and 15th runs were compared, mainly because those runs share similar extraction parameters of solid loading and time, but varied temperatures. Experiment run 15, which had an extraction temperature of 100 °C, extracted approximately 40% PUFAs, while the 5th run (at 60 °C) extracted only 11.7% of PUFAs. These results are in agreement with those reported previously [58], who found that the SFAs percentage decreased with increased microwave temperature of 120 °C. The PUFAs extraction increased at higher microwave temperature, attributable to the microwave efficacy in cell disruption, particularly at higher temperature. With increasing temperature, both mass and heat transport move in the same direction, from within the cell to the bulk solvent. Increased heating may have evaporated the water inside the cell, wherein the vapor introduces pressure that presses on the cell wall to cause cell rupture, which releases lipids either from inside the cell or from the cell wall itself [18]. PUFAs consider the main cell wall FAs component, which can be increased by increasing the number of ruptured cells.

**Figure 8:**
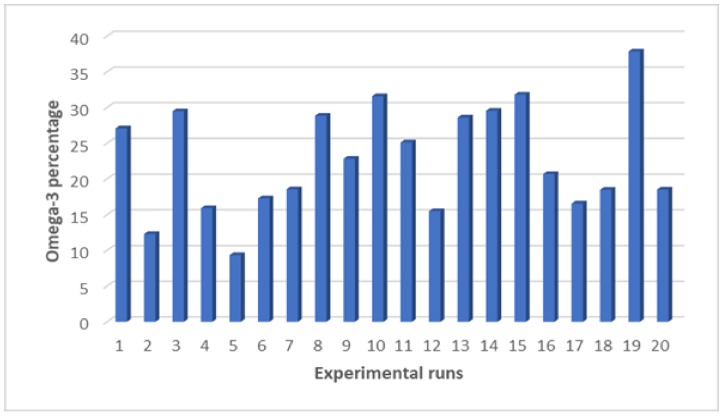
The distribution analysis for the experimental design including omega-3 percentage obtained from *Nannochloropsis sp*. microalgae via MAE technique with 10% (*w*/*v*) brine as the extraction solvent.

The most abundant FA among PUFAs is EPA, omega-3 FA. Figure 8 illustrates the changes in the distribution of omega-3 FAs, depending on the tested parameters. In general, it seems that the trend of omega-3 percentages is mostly influenced by temperature and time. When the extraction temperature and time increased, the EPA content increased, hence the increment in omega-3 percentage. As discussed above, the EPA is one of the most abundant compounds in *Nannochloropsis sp.* with the most influence on the PUFAs proportion.

The FAMEs amount and distribution for lipids extracted at optimum microwave parameters of 5% (*w*/*v*) solid loading, 100 °C temperature and 30 min extraction time using MAE-brine had been compared again with the conventional extraction results (Soxhlet and B&D). Utilizing the proposed method at optimum parameters resulted in significantly higher FAMEs amount (59 mg/g), when compared to that obtained from B&D method (38.7 mg/g) and superior to Soxhlet method (11.48 mg/g).

**Figure 9:**
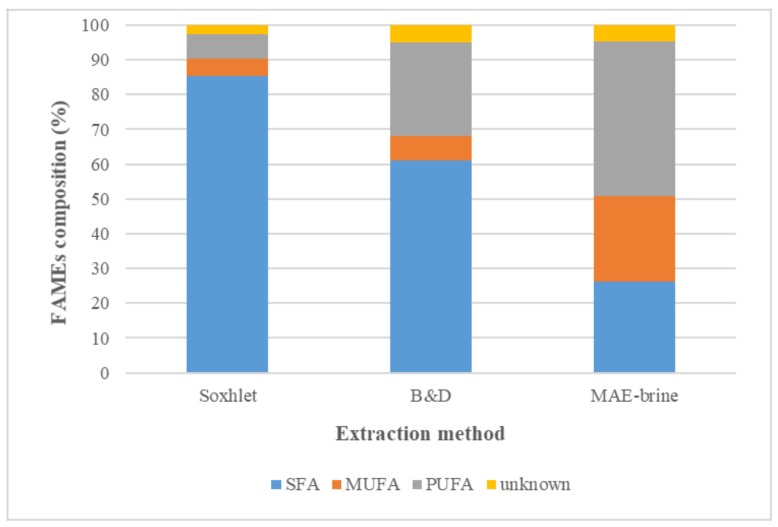
Comparison analysis of FAMEs distribution of *Nannochloropsis sp*. microalgae using different extraction methods of Soxhlet, B&D and MAE-brine at optimum extraction conditions.

Figure 9 clearly displays that MAE-brine at optimum extraction parameters extracted the highest percentages of PUFAs and MUFAs, while less percentage of SFAs. PUFAs were extracted approximately 44.5%, when compared to only 29.9% and 7.07% obtained using B&D and Soxhlet methods, respectively. The amount of extracted omega-3 was thrice higher (25.4 mg/g) than the amount obtained from B&D method (8.25 mg/g), while more outstanding than Soxhlet method (0.05 mg/g). These observations are crucial as they translate the benefits of employing the MAE method, along with 10% (*w*/*v*) brine as a green extraction solvent for lipid extraction from microalgae, so as to yield exceptional quality of edible lipid.

### 2.4. SEM Analysis

**Figure 10:**
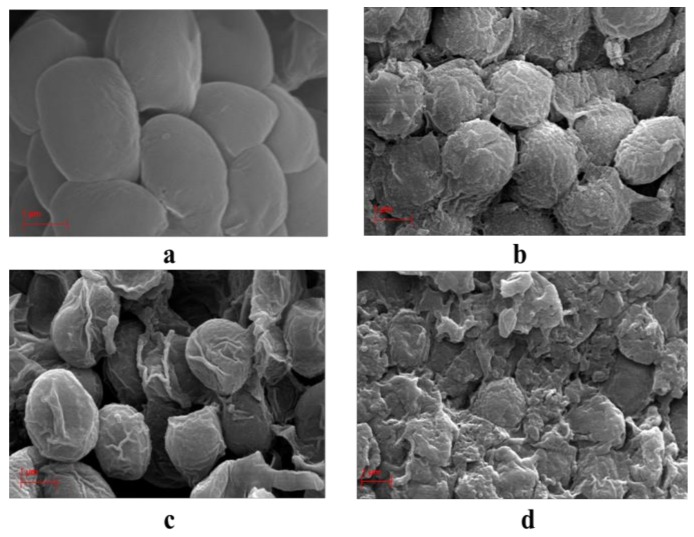
SEM micrographs (10K magnification) of the microalgal biomass before and after various lipid extraction methods: (**a**) original microalgal cell of Nannochloropsis sp., (**b**) microalgae cells after Soxhlet extraction, (**c**) microalgae cells after B&D method and (**d**) microalgae cells after MAE-brine technique at optimum extraction conditions.

In order to determine the structural impact of the extraction methods at the cellular scale, the morphologies of the microalgae cell were observed by SEM. Figure 10a–d shows the SEM pictures of *Nannochloropsis sp*. microalgae before and after conventional and MAE-brine extraction methods. It is clear that the whole cells following Soxhlet and B&D extraction in Figure 10b,c, respectively, are intact, similar to that of the untreated microalgae cells. The surface and size, however, have changed from smooth to the wrinkled surface and appear shrunken, especially after B&D extraction. Meanwhile, in Figure 10d, it is clear that the cell has disrupted, showing only debris of the cell walls. Thus, it is hypothesized that conventional and MAE methods follow different mechanism of lipid extraction. For Soxhlet and B&D methods, it could be deduced that the diffusion is the extraction mechanism. Meanwhile, the disruption is the main mechanism for the lipid extraction using MAE-brine technique.

It was found that the extraction of lipid in conventional techniques such as Soxhlet and B&D, follow the diffusion theory [59]. The lipid from the inside the cells diffuses through the membrane cell wall into the bulk solvent due to the affinity of extraction solvent toward the specific type of lipid. Since the cell walls are not disrupted, the diffusion mechanism is highly possible in the lipid extraction for the conventional methods where Soxhlet extraction involves a percolation of the extraction solvent (hexane) through the microalgae sample, while B&D extraction involves a suspension of microalgal species into the extraction solvent of chloroform and methanol. On the other hand, the cells expose to MAE showing broken cells, are likely to follow the disruption mechanism. Unlike conventional solvent extraction, the mass and heat transfer occur in the same direction in MAE (from inside to the outside of extracted material) [60]. This causes an internal superheating that leads to evaporation of the in-situ water of the microalgal cell. The severe evaporation creates a high pressure that leads to dramatic cell expansion and ruptures the cell wall. The PUFAs (especially EPA omega-3) are the main composition of *Nannochloropsis sp*. microalgal cell membrane. Thus, the efficiency of MAE in cell disruption has a direct impact on releasing these fatty acids. Moreover, the low extraction time of MAE can preserve the thermolabile compounds from degradation at a higher temperature.

The effect of the MAE technique on microalgae cell disruption and hence increasing on the extraction efficiency was reported previously, for example microwave irradiation resulted on 31% higher oil yield over conventional heating [61]. Another study found an increase in the bio-oil obtained from *Chlorella vulgaris* as a result of cell wall micro-cracks produced after treatment using MAE technique [62].

### 2.5. Economic Potential 

The extraction cost was estimated based on biomass, chemicals and electricity. The price for the calculations was of the purchased price at the quantity and quality for laboratory use only. In this study, the extraction efficiency has been presented in terms of yield (g lipid/100 g of microalgae). The cost required for producing 1 kg of lipid was calculated based on the lipid extraction efficiency for each extraction method, as shown in Table 6. The price of 1 kg of microalgae is USD 100.00 according to the supplier (Xi′an Lyphar Biotech Co., LTD, Xi′an City, China). More biomass is required (22.2 kg) as a result of low efficiency when using the Soxhlet method costing around USD 2220.00. The significantly lower number of microalgae were needed, for B&D and MAE-brine methods at 5.5 kg and 6.2 kg, respectively resulted in only USD 555.00 and USD 620.00, to produce 1 kg of lipid. As these methods require the use of solvents, the cost of chemicals used was also calculated. Although hexane is priced cheaper than chloroform at USD 6.00 versus USD 8.00 per liter, the high utilization of solvent in Soxhlet method resulted in enormous cost at nearly USD 8000.00 compared to around USD 825.00 in B&D method. The MAE-brine method shows the lowest chemical cost of USD 100.00 as a result of using cheap NaCl salt (R&M grade) and distilled water (taken as zero cost). The price of solvents and NaCl were provided by a local supplier (Evergreen Sdn. Bhd., Kuala Lumpur, Malaysia).

Both Soxhlet and MAE-brine techniques require the use of energy (calculated based on electricity cost). In this study, the power used to extract 5 g microalgae using Soxhlet was 180 W in 8 h (1.44 kWh). Meanwhile, it was 600 W and 0.5 h (0.3 kWh) for 2.5 g MAE-brine sample. The longer extraction time and lower lipid extraction efficiency in the Soxhlet method contributed to the higher energy cost for producing 1 kg of lipid. The electricity cost per 1 kWh was taken from the industrial rate of USD 0.073 according to Tenaga Nasional Berhad, Malaysia just for comparison purposes.

The total cost producing 1 kg of lipid for the different methods shows that the lowest operation cost is when the MAE-brine technique (USD 774.03), followed by B&D method, which is almost doubled (USD 1380.00). The tremendous cost was calculated for Soxhlet method (USD 10,683.99) mainly due to the high amount of solvent used for this process. It can be deduced that the solvent utilization is the main contributor for operating cost where the proposed MAE-brine technique used the least amongst the three methods.

## 3. Materials and Methods

### 3.1. Materials

The freeze-dried *Nannochloropsis sp.* full cell alga was purchased from Xi′an Lyphar Biotech Co., LTD, Xi′an, China. It was used as received, wherein the microalgae were cultivated at pH 8 using nitrate and phosphate (NP) with the concentrations of 1.6 g∙L^−1^ NaNO_3_ and 0.2 g∙L^−1^ NaH_2_PO_4_, respectively. Next, the biomass powder was vacuum-packed by the manufacturer until further use. The chemicals namely hexane, chloroform, methanol and sodium chloride were purchased from R&M Chemicals and of analytical grade (London WIV 3HP, Westminster, London, UK), while hydrochloric acid was bought from Sigma-Aldrich Co. (Sigma-Aldrich Co., Saint Louis, MO, USA).

### 3.2. Conventional Microalgae Lipid Extraction

#### 3.2.1. Soxhlet Extraction

Five grams of dried *Nannochloropsis sp.* algae was transferred to cellulose extraction thimble (Whattman, 28 mm id × 80 mm length). The lipids were extracted into 300 mL hexane. The solvent was heated to the boiling point of hexane (~65 °C) and refluxed approximately 20 times per hour for 8 h using the standard Soxhlet apparatus. The lipids were determined gravimetrically after they were recovered by drying off the extracting solvent. Then the yield was calculated according to Equation (6). The experiment was carried out in triplicates.

(6) $$\mathrm{Lipid}\;\mathrm{yield}\;(\%)\;=\;\frac{mass\;of\;extracted\;lipid\;(g)}{mass\;of\;dried\;microalgae\;(g)}\;\times \;100\%$$

#### 3.2.2. Bligh and Dyer Extraction 

Lipid extraction was performed via modified procedures described by Bligh and Dyer [13]. Two grams of dried microalgae was mixed with 8 mL of distilled water. Next, 38 mL of solvent mixture (chloroform/methanol/water) at a ratio of 1:2:0.8 (*v*/*v*) was added and homogenized using vortex mixer (Thermo Scientific LP, Nashville, TN, USA) for 5 min. A mixture of chloroform and water 1:1 (*v*/*v*) ratio was added to form a two-phase system.

The two phases were separated by centrifugation at 3500 rpm for 5 min. The lower phase, which contains lipids, was gently withdrawn using a Pasteur pipette to avoid contamination at the other phase. When the chloroform solvent had evaporated, the lipid was measured gravimetrically and the yield was calculated according to Equation (6). The experiment was carried out in triplicates.

### 3.3. Microwave-Assisted Extraction (MAE)

#### 3.3.1. Microwave Extractor Setup

**Figure 11:**
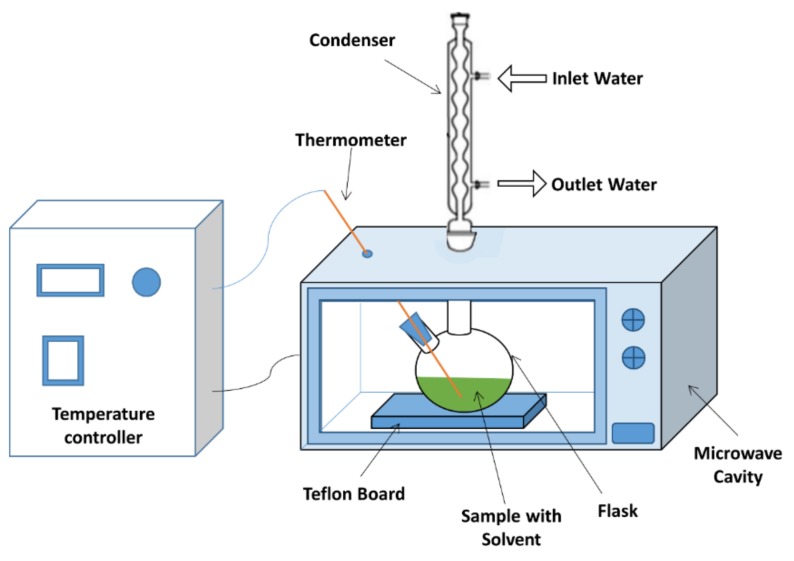
Schematic drawings of the MAE system.

The MAEs were performed in a modified domestic microwave (Samsung, ME711K) combined with a temperature control device that had a working temperature ranging between 20 and 240 °C. The microwave had a maximum output of 800 Watt that operated at a frequency of 2.45 GHz. The top of the microwave cavity had two 25 mm round holes, with the center hole connecting the round bottom flask with the condenser cooled by tap water, while the side hole facilitating the installment of thermocouple in the solvent. A Teflon board was placed at the bottom of the flask to ensure no arching during microwave runs. Both timer and temperature were controlled via the control system attached to the microwave oven. Figure 11 illustrates the schematic drawing of the microwave system.

#### 3.3.2. Experimental Procedures

The microwave extraction was performed in 250 mL round bottom borosilicate flask. Different sets of experiments were run for MAE in triplicates. In order to investigate the effect of MAE solvent on lipid and PUFA yield, a predetermined amount of inorganic NaCl salt was dissolved in 50 mL distilled water to obtain concentrations ranging from 1 to 35% (*w*/*v*) to function as solvent. Five g of *Nannochloropsis sp.* microalgae powder sample was added to the salt solution to get 10% (*w*/*v*) solid loading. Pure distilled water was also used as solvent as a comparison experiment (named 0%). The microwave was run at 100 °C for 5 min continuously, while the condenser was cooled under flowing tap water.

As for the optimization experiments, varied amounts of dried *Nannochloropsis sp.* powder sample were added to the optimum salt solution at different temperatures and times, depending on the designed experiment. Section 3.3.3 elaborates the experimental runs.

#### 3.3.3. Optimization Experiment and Statistical Analysis 

In this experiment, the effects of several microwave factors on lipid extraction were tested. The RSM was applied for this purpose. A Central Composite design (CCD) with three factors and five levels (−α,−1,0,+1,+α) was employed for the RSM experiment. The three extraction factors comprised of solid loading (A), temperature (B) and time (C). Table 7 lists the required matrix for CCD, including the actual and coded levels. Based on the CCD experimental design, 20 runs were performed.

ANOVA analysis was performed to assess the effects of microwave extraction factors, as well as the interaction between them in light of response factor (lipid yield). The behavior of each variable in predicting the response factors was demonstrated in the following equation:

Y = β_0_ + β_1_A + β_2_B + β_3_C + β_12_AB + β_13_AC + β_23_BC + β_1_^2^A^2^ + β_2_^2^B^2^ + β_3_^2^C^2^ (7)

where Y is the predicted response factor; A, B, and C are independent variables, β_0_ reflects the intercept, whereas (β_1_, β_2_ and β_3_), (β_12_, β_13_ and β_23_) and (β_12_, β_22_ and β_32_) are linear coefficients, interaction coefficients and quadratic coefficients, respectively. In order to examine the adequacy of the model and to obtain the final equation model, the statistical significance for each factor in Equation (7) was determined. The statistical significance mainly depends on *p*-value less than 0.05 (*p* < 0.05). To illustrate the statistical significance described by ANOVA, the Standardized Pareto chart was applied. Finally, response surface graphs were acquired as a result of individual or combinatory effect(s) of the independent variables.

The optimum values for each independent variable were obtained from RSM analysis. In verifying the outcomes, lipid extraction from *Nannochloropsis sp*. microalgae was conducted thrice at the optimum level of independent variables.

#### 3.3.4. Samples Post-treatment

After the microwave extraction procedure, a few ml (~5 mL) of hexane had been added to the treated samples to recover the extracted lipid into non-polar organic layer. The upper hexane layer was recovered and dried after the mixture was centrifuged at 3500 rpm for 5 min. The weight of the lipid was recorded and the lipid was stored in sealed glass vials at –20 °C for further analysis.

### 3.4. Transesterification of Lipid

The FAMEs were prepared from the extracted *Nannochloropsis sp.* lipids via transesterification reaction following the method prescribed by [63]. First, the extracted lipids were vortexed for 15 s with 3 mL of mixture containing CH_3_OH/HCl/ CHCl_3_ (10:1:1 *v*/*v*/*v*). After that, the samples were heated at 90 °C for an hour.

Once the transesterification reaction was completed and the sample was cooled to room temperature, 1 mL of distilled water was added and the mixture was vortexed. Next, 2–3 mL mixture of C_6_H_12_/CHCl_3_ (4:1 *v*/*v*) was added to form two layers. The upper organic layer, which contained FAMEs fraction, was recovered, filtered and evaporated to determine FAME yield, see Equation (8). Prior to the GC analysis, the FAMEs fraction was dissolved in 1.5 mL of hexane and was transferred into sealed GC vials.

(8) $$\mathrm{FAMEs}\;\mathrm{yield}\;(\mathrm{mg}/g)\;=\;\frac{mass\;of\;extracted\;FAMEs\;(mg)}{mass\;of\;dried\;microalgae\;(g)}$$

### 3.5. FAME Analysis Using GC-FID 

FAMEs were separated and identified using GC (Agilent 6890 GC, Saint Paul, MN, USA) equipped with FID. The 30-m length, 0.25-mm diameter and 0.25-μm film thickness polar ZB-WAX column (Zebron, Newport, CA, USA) was applied to disintegrate FAME. The hydrogen gas carrier was flowed at a rate of 3 mL/ min. Two µl samples were injected at an inlet temperature of 250 °C using splitless mode. The initial oven temperature was 100 °C, and after a minute, the oven temperature was ramped to 230 °C at 5 °C/min and maintained for 5 min at the final temperature. Meanwhile, the detector temperature was set at 260 °C. The qualification and quantification analyses for 20 FAMEs were carried out by comparing each FAME retention time with the standard FAME mixture supplied by Marine Oil FAME Mix (Restek, Bellfonte, PA, USA).

### 3.6. Scanning Electron Microscopy

The Scanning Electron Microscopy (SEM) analysis was implemented on *Nannochloropsis sp.* microalgae before and after different extraction methods of Soxhlet, B&D, and MAE-brine at different extraction temperature and time. The microalgal sample was stabilized/fixed with 3% glutaraldehyde buffered with 0.1 M phosphate buffer. The sample was stabilized for 2 h. After several times washing with 0.1 M phosphate buffer, the mixture was immersed in 1–2% osmium tetroxide in buffer solution (pH 7.2) for another 2 h. After 2 h, the mixture was dehydrated in acetone prior to being dried with CO2 at critical point using critical point dryer (BAL-TEC, S00111087, CA, USA). Then the dried samples were mounted on SEM stubs and coated with gold in the Edwards vacuum (BAL-TEC Bai-Tec Scd 005, CA, USA). After 15 min, the coated sample can be imaged using SEM instrument (HITACHI, S-34OON, Chiyoda-ku, Tokyo, Japan).

## 4. Conclusions

The MAE method for lipid and PUFAs extraction from *Nannochloropsis sp.* microalgae was carried out using economic and green solvent. The effect of salt concentration on the extraction yield was investigated, whereby the highest quantity of lipids yield and the best quality of FAMEs were achieved at 10% (*w*/*v*) salt concentration. The optimum microwave extraction conditions were estimated to be 5% (*w*/*v*) solid loading, 100 °C and 30 min extraction time. Comparison with conventional methods exemplified that lipid yield via optimized MAE-brine (16.1%) was slightly lower than that via B&D method (18%), but superior to the Soxhlet method (4.5%). The extracted lipids using MAE-brine technique seemed to contain higher amount of PUFAs, with more omega-3, thus signifying outstanding quality.

## Figures and Tables

**Table 1:** Percentage of chemical composition for various microalgae (% dry biomass) [3,4].

Microalgae	Protein	Carbohydrate	Lipids
*Anabaena cylindrical*	43–56	25–30	4–7
*Chlamydomonas rheinhardii*	48	17	21
*Chlorella vulgaris*	38	33	5
*Diacronema vlkianum*	57	32	6
*Dunaliella salina*	39–61	14–18	14–20
*Haematococcus pluvialis*	48	27	15
*Isochrysis galbana*	50–56	10–17	12–14
*Scenedesmus obiquus*	6–20	33–64	11–21
*Spirulina maxima*	46–63	8–14	4–9
*Spirulina platensis*	52	15	3
*Nannochloropsis sp.*	64	3	23

**Table 2:** Summarization of recent studies that comparing microwave-assisted extraction (MAE) with other non-conventional methods for lipid extraction yield from different microalgal species.

Microalgae	Extraction Method	Solvent	Lipid Yield (%)	References
*Botryococcus sp.,* *Chlorella vulgaris,* *Scenedesmus sp.*	Autoclave Microwave Ultrasound	Chloroform/ methanol (1:1 *v*/*v*)	5.4–11.9 10–28.6 6.1–8.8	[10]
*Nannochloropsis sp.*	Microwave Ultrasound	Chloroform/ methanol (2:1 *v*/*v*)	32.8 18.9	[25]
*Chlorella sp.*	Microwave Autoclave	Chloroform/ methanol (2:1 *v*/*v*)	38 24	[26]
*Mixed culture*	Microwave Autoclave Ultrasound Electroflotation	chloroform/ methanol/ water (1:2:0.8 *v*/*v*)	33.7 15.4 13.3 24.8	[27]
*Chlorella vulgaris*	Grinding Ultrasound beadmilling Microwave	Chloroform/ methanol (1:1 *v*/*v*)	6 15 10 18	[28]
*N. gaditana*	Microwave Ultrasound	Chloroform/ methanol/ water (1:2:1 *v*/*v*/*v*)	39 36	[29]
*C. vulgaris*	Ultrasound Microwave	Chloroform/ methanol/ water (2:2:1.8 *v*/*v*/*v*)	26.4 28.9	[30]

**Table 3:** The fatty acid methyl esters (FAMEs) composition of *Nannochloropsis sp*. microalgae (in mg/g) at different salt concentrations (0%–35%) using MAE at 10% (*w*/*v*) solid Loading, 100 °C and 5 min.

FAs	FAMEs (mg/g)								
	0%	1%	5%	10%	15%	20%	25%	30%	35%
C14:0	0.26	0.63	1.10	4.02	1.88	2.97	1.46	1.21	0.80
C14:1	0.01	0.00	0.03	0.31	0.16	0.22	0.13	0.14	0.08
C16:0	1.12	2.97	7.10	9.19	13.95	4.68	9.51	7.60	5.51
C16:1	0.92	1.24	0.71	0.51	0.82	0.59	0.16	1.14	0.03
C18:0	0.00	0.04	0.08	0.25	0.12	0.25	0.89	0.08	0.07
C18:1	0.24	0.11	0.60	1.14	0.97	2.42	0.15	0.72	0.43
C18:1	0.04	0.29	0.34	1.64	0.55	0.80	0.37	0.08	0.06
C18:2	0.01	0.10	0.24	0.13	0.00	0.87	0.37	0.32	0.17
C18:3	0.01	0.06	0.04	0.30	0.09	0.16	0.08	0.05	0.03
C20:0	0.00	0.02	0.00	0.06	0.05	0.08	0.05	0.04	0.05
C20:1	0.00	0.00	0.00	0.12	0.05	0.08	0.06	0.05	0.00
C20:2	0.00	0.02	0.00	0.62	0.03	0.00	0.00	0.06	0.03
C20:4	0.04	0.00	0.27	1.16	0.68	0.93	0.39	0.39	0.17
C20:3	0.00	0.12	0.02	0.10	0.14	0.15	0.03	0.05	0.04
C20:5	0.14	0.45	1.19	8.15	2.87	4.02	1.78	1.71	0.64
C22:0	0.03	0.04	0.03	0.34	0.21	0.03	0.07	0.12	0.08
C22:1	0.03	0.05	0.15	0.68	0.28	0.24	0.15	0.12	0.18
C24:0	0.03	0.05	0.04	0.00	0.00	0.42	0.27	0.17	0.10
C22:6	0.00	0.00	0.00	0.09	0.03	0.06	0.03	0.00	0.00
C24:1	0.03	0.06	0.08	0.03	0.15	0.23	0.03	0.04	0.06
Unknown	0.11	0.22	0.34	0.58	1.46	1.20	0.47	0.36	0.51
SFA	1.44	3.75	8.35	13.86	16.21	8.44	12.26	9.22	6.61
MUFA	1.28	1.76	1.91	4.41	2.98	4.58	1.05	2.29	0.84
PUFA	0.18	0.65	1.52	10.42	3.84	5.31	2.31	2.26	0.91
Omega-3	0.15	0.51	1.23	8.54	2.99	4.24	1.89	1.76	0.67

**Table 4:** The matrix of Central Composite Design (CCD) with experimental lipid yield values of MAE.

Runs	Coded Parameters			Process Parameters			Lipid Yield (%)
	A	B	C	Solid Loading (%)	Temperature (°C)	Time (min)	
1	−1	−1	+1	10	70	25	5.10 ± 0.04
2	−1	−1	−1	10	70	10	3.91 ± 0.02
3	+1	+1	−1	20	90	10	5.03 ± 0.01
4	0	0	−α	15	80	5	3.84 ± 0.10
5	0	−α	0	15	60	17.5	2.10 ± 0.11
6	0	0	0	15	80	17.5	5.42 ± 0.03
7	0	0	0	15	80	17.5	3.18 ± 0.03
8	−1	+1	−1	10	90	10	9.30 ± 0.03
9	+1	−1	+1	20	70	25	2.64 ± 0.05
10	−1	+1	+1	10	90	25	9.95 ± 0.02
11	0	0	0	15	80	17.5	3.2 ± 0.03
12	−α	0	0	5	80	17.5	8.75 ± 0.10
13	+α	0	0	25	80	17.5	2.56 ± 0.04
14	0	0	+α	15	80	30	5.47 ± 0.03
15	0	+α	0	15	100	17.5	7.68 ± 0.12
16	0	0	0	15	80	17.5	3.87 ± 0.03
17	+1	−1	−1	20	70	10	2.43 ± 0.10
18	0	0	0	15	80	17.5	3.77 ± 0.02
19	+1	+1	+1	20	90	25	7.81 ± 0.02
20	0	0	0	15	80	17.5	2.82 ± 0.04

**Table 5:** ANOVA statistical analysis for CCD results.

Source	Sum of Squares	Df	Mean Square	F-Ratio	*P*-Value
Model	106.65	9	11.85	13.25	0.0002
**Linear**					
A	31.57	1	31.57	35.31	0.0001
B	54.90	1	54.90	61.40	<0.0001
C	4.20	1	4.20	4.70	0.0555
**Interaction**					
AB	0.76	1	0.76	0.85	0.3785
AC	0.16	1	0.16	0.18	0.6777
BC	0.51	1	0.51	0.58	0.4656
**Quadratic**					
A^2^	9.64	1	9.64	10.79	0.0082
B^2^	4.36	1	4.36	4.87	0.0518
C^2^	3.11	1	3.11	3.48	0.0918
Residual	8.94	10	0.89		
Lack of Fit	4.65	5	0.93	1.08	0.4662
Pure Error	4.29	5	0.86		
Cor Total	115.60	19			

R^2^ = 0.9227.

**Table 6:** The cost assessment for production 1 kg of lipids from *Nannochloropsis sp.* microalgae using different extraction methods.

Economic Evaluations	Extraction Method Costs (USD)		
	Soxhlet	B&D	MAE-Brine
Microalgae biomass	2220.00	555.00	620.00
Chemicals	8000.00	825.00	100.00
Electricity	463.99	Not applicable	54.03
Total	10,683.99	1380.00	774.03

**Table 7:** Variables and levels involved in CCD experiment.

Variables		Levels				
Coded	Actual	−α	−1	0	+1	+α
A	Solid loading (%)	5	10	15	20	25
B	Extraction temperature (°C)	60	70	80	90	100
C	Extraction time (min)	5	10	17.5	25	30
